# Supplemental Herbal Choline Increases 5-hmC DNA on Whole Blood from Pregnant Ewes and Offspring

**DOI:** 10.3390/ani10081277

**Published:** 2020-07-27

**Authors:** José Alejandro Roque-Jiménez, German David Mendoza-Martínez, Anayeli Vázquez-Valladolid, María de la Luz Guerrero-González, Rogelio Flores-Ramírez, Juan Manuel Pinos-Rodriguez, Juan J. Loor, Alejandro Enrique Relling, Héctor Aarón Lee-Rangel

**Affiliations:** 1 Facultad de Agronomía y Veterinaria, Universidad Autonoma de San Luis Potosí, Carretera Federal 57 Km 14.5, Ejido Palma de la Cruz, Soledad de Graciano Sánchez, San Luis Potosí 78321, Mexico; alejandro_roque@alumnos.uaslp.edu.mx (J.A.R.-J.); anayeli.vazquez@uaslp.mx (A.V.-V.); luz.guerrero@uaslp.mx (M.d.l.L.G.-G.); 2 Departamento de Producción Animal, Universidad Autonoma Metropolitana—Xochimilco, CDMX, Mexico City 04960, Mexico; gmendoza@correo.xoc.uam.mx; 3 Centro de Investigación Aplicada en Ambiente y Salud, CIACYT—Medicina, Universidad Autonoma de San Luis Potosí, Lomas de San Luis 78210, Mexico; rogelio.flores@uaslp.mx; 4 Facultad de Medicina Veterinaria y Zootecnia, Universidad Veracruzana, Veracruz 91710, Mexico; jpinos@uv.mx; 5 Department of Animal Sciences, Division of Nutritional Sciences, University of Illinois, 262 Animal Sciences Laboratory, Urbana, IL 61801, USA; jloor@illinois.edu; 6 Department of Animal Science, The Ohio State University, Ohio Agricultural Research and Development Center (OARDC), Wooster, OH 44691, USA; relling.1@osu.edu

**Keywords:** sheep, early feeding, epigenetic changes

## Abstract

**Simple Summary:**

DNA hydroxymethylation (5-hmC) is an epigenetic mechanism that modifies the five positions of cytosine through the addition of a hydroxymethyl group to DNA. In the last decade, the use of herbal products, marketed as dietary supplements or “nutraceuticals” in some countries, has increased rapidly; however, there is a lack of evidence on the extent to which formulas used during pregnancy cause epigenetic changes in the fetus. The aim of this study was to characterize the effects of supplementing an herbal choline source (BCho) on 5-hmC DNA in whole blood from gestating ewes and their offspring. Such data would provide evidence of nutritional programming effects.

**Abstract:**

Herbal formulas during pregnancy have been used in developing countries. Despite that, the potential effects on the mother and offspring and whether those supplements elicit epigenetic modifications is still unknown. Therefore, our objectives were to determine the effects of supplemental herbal choline source (BCho) on the percentage of 5-hmC in whole blood from gestating ewes and their offspring, as well as determining the milk quality and growth of the offspring. Thirty-five gestating Rambouillet ewes were randomly assigned to five treatments: T1, supplementation of 4 g per day (gd^−1^) of BCho during the first third of gestation; T2, supplementation of 4 gd^−1^ of BCho during the second third of gestation; T3, supplementation of 4 gd^−1^ of BCho during the last third of gestation; T4, supplementation of 4 gd^−1^ of BCho throughout gestation; and T5, no BCho supplementation (control). For the 5-hmC DNA analysis, whole blood from ewes was sampled before pregnancy and at each third of gestation (50 days). Whole blood from lambs was sampled five weeks after birth. The evaluation of the nutritional programming effects was conducted through the percentages of 5-hmC in the lambs. Compared with other treatments, the whole blood from ewes supplemented during T1 and T4 had the greatest 5-hmC percentages (*p* < 0.05). However, only ewes fed BCho throughout gestation (T4) maintained the greatest percentages of 5-hmC (*p* < 0.05). The lamb growth performance indicated that the BCho maternal supplementation did not affect the nutritional programming. However, the lambs born from ewes supplemented during T2 had the greatest 5-hmC percentages (*p* < 0.05). Our data suggest that ewes supplemented during T4 with BCho increase and maintain the percentages of 5-hmC in whole blood, and the offspring born from ewes supplemented with BCho during T2 maintained the greatest percentages of 5-hmC 35 d after they were born.

## 1. Introduction

Maternal nutrition during pregnancy changes the physiological and metabolic phenotypes of the offspring [1], with researchers suggesting an adaptation by the fetus known as fetal developmental, offspring health, and growth during childhood [2]. Nutritional programming can affect the offspring health, fetal development, and growth during adulthood [3]. The early postnatal period is a critical period in which nutrition can program the offspring [4]. Physiological and metabolic mechanisms are not fully mature at birth and continue to develop in the immediate postnatal period [5]. Some authors reporting the effects of maternal nutrition during pregnancy on offspring development might be confounded with post-natal growth during lactation. The reason for this is because the mammary gland function and colostrum yield are also impacted by nutritional management prepartum [6]. Early postnatal life has shown to be another critical window for metabolic programming during which nutrition can induce epigenetic changes [7]. For instance, lambs immediately separated from their dams and fed artificial colostrum had a lower body weight, and their levels of IgG were measured 24 h later. The offspring from undernourished dams had increased IgG transfer compared with those of control-fed dams [8]; this suggests that the fetal gastrointestinal system may be programmed in nutrient-restricted animals with a base in colostrum and milk. Studies in beef cattle revealed that maternal nutrient restriction during late gestation reduces the postnatal calf birth weight [9]. Clearly, these data underscore the role of maternal nutrition on offspring growth. Nevertheless, there is still controversy over the efforts to understand fetal nutrition, and there is a lack of evidence in ruminants regarding developmental programming effects [10]. Herbal feed additives or supplements contain different bioactive compounds, including polyphenols, isothiocyanates, saponins, and terpenoids, all of which can modulate the epigenome through interactions with polycomb groups and methyl-CpG binding proteins [11]. Herbal additives such as BCho have been tested in different farm animals. Crosby et al. [12] observed that supplementation with 4 gd^−1^ of BCho from 30 d before to 30 d after parturition in ewes increased lamb birth weight, milk yield, and the oleic acid content in milk. In addition, supplementing 4 gd^−1^ of BCho to lambs during the finishing period alters the mobilizing of non-esterified fatty acids, stimulating glucose and cholesterol synthesis [13]. Additionally, Martinez-Aispuro et al. [14] observed that the inclusion of BCho improved the daily weight gain and feed efficiency of finishing lambs. In dairy cattle, Gutiérrez et al. [15], using 17 gd^−1^ of BCho for three years, observed a reduction in abortions, clinical and subclinical mastitis, respiratory disorders, and hypocalcemia incidence. Mendoza et al. [16] concluded that the use of 15 gd^−1^ of BCho in Holstein cows increases milk production; however, the supplementation with BCho did not modified the milk composition (fat, lactose, total solids, and non-fatty solids). Despite these improvements in animal production, the mechanisms behind these changes have not been elucidated.

The outcomes from feeding herbal formulas may also depend on the principles of transgenerational epigenetics, whereby a connection between individuals and their local environment, including exposure to local weather, nutrition, and health, renders them susceptible to epigenetic changes that favor individual development, pregnancy, and the offspring [17,18]. The mechanisms by which maternal nutrition can affect fetal development have been linked with epigenetic marks regulating gene expression without affecting the DNA sequence. During pregnancy, there is an increase in the methyl-group donor requirements, such as folate, betaine, choline, methionine, and free methyl groups. These methyl-group donors play a central role in the one-carbon metabolism pathway. The one-carbon metabolism is central in the methylation of all biological molecules, including DNA methylation, histone modifications [18], as well as the activity of microRNAs and long non-coding RNAs [7,8].

One of the underlying mechanisms responsible for fetal and nutritional programming is epigenetic modifications, including DNA hydroxymethylation (5-hmC) [19]. These 5-hmC marks are considered a transient intermediate in DNA demethylation, but have recently been highlighted as a stable epigenetic mark [19,20]. The 5-hmC marks determine methylation status throughout the whole genome [20]. Thus, they might participate in the process of differentiation or cellular development [19,21].

Shortly after conception, the maternal and paternal DNA are globally demethylated, which is followed by de novo methylation just before implantation. This is a critical window in fetal development during pregnancy where dietary factors can influence the methylome [22]. The mechanism of how DNA methylation and 5-hmC marks change during different stages of pregnancy is still unknown. Whether maternal methyl-group-supplemented diets affect the offspring epigenome in livestock species also is unclear. Work in model organisms confirms the biological possibility of the nutritional programming of the fetus in response to the maternal supply of nutrients capable of causing epigenetic effects (i.e., methyl donors) [23].

We hypothesized that the herbal compounds of herbal choline source (BCho) have epigenetic proprieties specifically in the context of 5-hmC and can impact the fetal development and offspring growth in sheep. Therefore, the objectives were (1) to characterize the natural compounds BCho by gas chromatography coupled with mass spectrometry (GC-MS), (2) to determine the effect of BCho supplementation on the 5-hmC in whole blood from gestating ewes and their offspring, and (3) to evaluate and describe the effect of BCho on the programming of the offspring early in life through the evaluation of the colostrum and milk quality.

## 2. Materials and Methods

### 2.1. Ethics

The animal procedures were reviewed and approved by the Committee for the Ethical Use of Animals in Experiments of the Universidad Autonoma de San Luis Potosi, according to the regulations and standards that are required by the Mexican government for the use of animals for a number of diverse activities. Federal law on technical specifications for the care and use of laboratory animals and for livestock farms; farms; centers of production, reproduction, and breeding; zoos; and exhibition halls must meet the basic principles of animal welfare (NOM-062-ZOO-1995).

### 2.2. Location

The research was conducted at the sheep facilities of the Facultad de Agronomía y Veterinaria of Universidad Autónoma de San Luis Potosí, Soledad de Graciano Sánchez, San Luis Potosí, México (Latitude 22°14′0.58″; Longitude 100°50′48.5″), from December 2018 to June 2019.

### 2.3. Choline Characterization

The herbal choline source (BCho; Biocholine Powder^®^, Nuproxa, LTD, Switzerland) is a commercial herbal formula labeled to contain 16 g/kg of phosphatidylcholine ((PCho), natural choline conjugates), equal to 1.6 mg/kg of PCho, to ensure 64 mg/d of PCho per ewe. The extraction of bioactive compounds was performed using an ultrasonic processor (GEX130, 115 V 50/60 Hz) equipped with a 3 mm titanium tip and mechanical stirrers (Cole-Parmer, IL, USA). One gram of BCho was mixed with 10 mL of acetone. Subsequently, the organic phase was separated, concentrated to 1 mL of extracted mixture, and evaporated (Zymark, Turbovap LV Concentration Evapotarot, NB, USA) for the final analysis.

The characterization of BCho was performed with gas chromatography (GC-HP 6890) coupled with mass spectrophotometry (MSHP 5973), equipped with a capillary column 60 m length, 0.255 mm diameter, and 0.25 µm film thickness (HP 5MS, Agilent). The temperature program was 70 °C for 2 min, which was then increased to 250 °C at the rate of 20 °C/min, then to 290 °C at the rate of 5 °C/min, then increased to 300 °C at the rate of 1 °C/min, then to 310 °C at the rate of 5 °C/min and kept there for 36 min. The injector temperature was 250 °C in splitless mode. The helium flow rate was 1 mL/min. The mass spectrophotometry was programed in SCAN mode (50–500 m/z) to identify compounds.

### 2.4. Animals, Treatments, and Sampling

Forty 3-year-old Rambouillet ewes with previously one parturition (initial body weight (IBW) 51.5 kg ± 0.5) were assigned randomly to five treatments. All the ewes were bred by a single Rambouillet ram (IBW 74.5 kg). The ram used a marking harness with paint during the breeding. Immediately after mating, each of the ewes were housed in an individual pen and randomly assigned to a treatment. The day of the mating was considered day one of conception. Twenty-eight days after the mating, ultrasonography was used confirm pregnancy. Accordingly, five non-pregnant ewes were removed from the groups. Finally, 35 pregnant ewes (7 ewes for treatment) were assigned to one of five treatments: (T1) supplementation of 4 gd^−1^ of BCho during the first third of gestation (day 1 to day 49); (T2) supplementation of 4 gd^−1^ of BCho during the second third of gestation (day 50 to day 99); (T3) supplementation of 4 gd^−1^ of BCho during the last third of gestation (day 99 to day 149); (T4) supplementation of 4 gd^−1^ of BCho throughout gestation; and (T5) no BCho supplementation (control) in a completely randomized design. The target amount of BCho supplementation was 0.0724 gd^−1^ choline conjugates. Previous studies have demonstrated that this dose changes the lamb birth weight and milk yield and decreases the incidence of disease [12,13,14,15]. The supplementation with BCho was top-dressed on the feed. The ewes were fed corn silage (Table 1). The corn silage samples were collected weekly, pooled, and analyzed according to AOAC [24] for dry matter (DM, method number 981.10), crude protein (CP, method number 967.03), neutral detergent fiber (NDF), and acid detergent fiber (ADF) according to Van Soest et al. [25] with a heat-stable amylase included in the NDF.

Whole blood was sampled from the ewes in 4 different periods: 10 days before conception, and 50, 100, and 150 days after conception at 08:00 h (1 h before feeding). Whole blood samples from the lambs were collected 5 weeks after birth in the morning. Two 3 mL samples per animal were taken from the jugular vein of ewes and lambs and immediately transferred to a DNA Shield blood collection tube (Zymo Research, USA) and placed in liquid nitrogen. After collection, the samples were stored at −80 °C until further analysis.

### 2.5. DNA Isolation

The whole blood was purified and the DNA isolated using a Genomic Lysis Buffer^TM^ (Mexico City, Mexico) for biological and solid samples. The extraction of genomic DNA was performed using a commercial kit with spins (D3024 Zymo-Spin^TM^ IIC Column, Zymo Research, Irvine, CA, USA) according to the manufacturer´s protocol. The extracted DNA was quantified using UV spectroscopy (Epoch^TM^ 2 Microplate, Biotek, Winooski, VT, USA) and qualitatively assessed using a 0.8% agarose gel stained with ethidium bromide and confirmed via gel electrophoresis on a transilluminator (Benchtop 2UV^TM^, Cambridge, UK).

### 2.6. Quantification of 5-hidroxymethylation (5-hmC)

The whole blood genomic DNA 5-hmC percentages were determined by a colorimetric ELISA using the Quest 5hmC DNA ELISA kit (Zymo Research, USA). The whole blood genomic and control DNA were analyzed simultaneously, and different batches of the ELISA kit were used to analyze all the DNA samples, thereby limiting the variation in measurements resulting from potential procedural or batch discrepancies. The assays were performed according to the manufacturer´s instructions, loading 100 ng of DNA per well. The absorbance at 405 nm was captured using an Epoch^TM^ 2 microplate reader (Biotek, Winooski, VT, USA). The percentage of 5-hmC is expressed as the mean + the standard error of the mean (SEM).

### 2.7. Nutritional Programming Analyses

All the ewes in the study had one rearing during the births. The total mating time was 5 consecutive days. The ewes gave birth under similar weather (temperature averages 32 °C maximum and 11 °C minimum during the test). After birth, each ewe remained housed in individual pens with her offspring until the end of the experiment. The lambs were weighed on day 1 (lambing), and five weeks later (35 days old). All the lambs survived the entire experimental period. The daily weight gain (DWG) was estimated for day 1 to 35. Ten milliliters of colostrum were obtained manually at the moment of delivery, placed into cryogenic vials (Thermo Fisher Scientific, Waltham, MA, USA), and then stored at −20 °C until analysis. The milk production was measured each 7 days, as described by Reynolds et al. [26]; the composition was measured on day 35. Briefly, the ewes were separated from their kids at 07:00 h and immediately milked by hand; this milk was offered to the lamb kids. After 3 h, an injection of oxytocin (20 IU) into the jugular vein was administrated, and after this hand milking was repeated; the yield was recorded by the milk sample collected. The milk samples were frozen at −20 °C until further analysis. Each sample of colostrum was put in a water bath for 1 min (40 °C), homogenized until the colostrum temperature reached 29 °C, and analyzed according to the manufacturer’s protocol for colostrum in a Lactoscan Ultrasonic Milk Analyzer (Milkotronic, Nova Zagora, Bulgaria). For the milk analysis, each sample was previously mixed and homogenized in a water bath for 1 min (40 °C) until the milk temperature reached 29 °C; after this, the samples were analyzed according to the manufacturer’s protocol for milk in the same equipment. For the colostrum and milk analysis, none of the samples were diluted.

### 2.8. Statistical Analysis

The experimental design was a completely randomized design. The data were analyzed with the MIXED procedure of SAS (9.4 SAS Inst. Inc., Cary, NC, USA), where the ewes were a random component and the treatments were the fixed components in the model. The measurements through time were analyzed as repeated measures, adding the effect of time and treatment by time interaction as fixed components. Because there was not a treatment by time interaction (*p* > 0.10) for any of the variables, only the main effect of treatment is presented in the manuscript. The PDIFF option of SAS was used for mean separation. A probability of ≤0.05 was considered statistically significant. The data are presented as LS means and the standard errors of the mean (SEM).

## 3. Results

### 3.1. Biocholine Characterization

**Figure 1:**
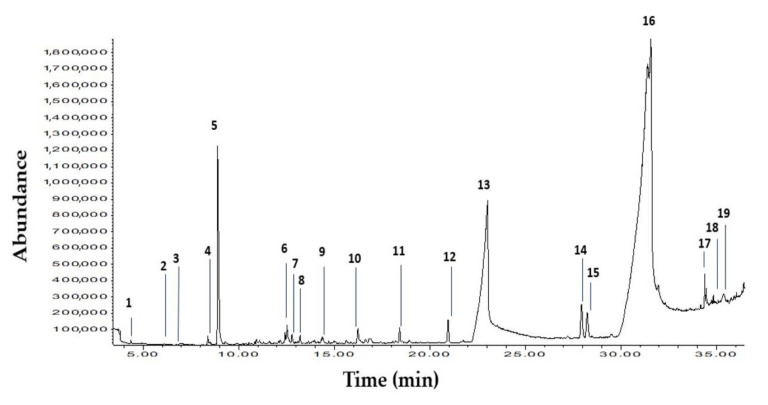
Total ion chromatogram of the volatile components and chemical composition in BCho. Chemical composition of Biocholine (BCho) by CG-MS with retention time^(rt)^: 1. 6-Undecanol; 2. 2-Methyl-2-octene; 3. 4H-Pyran-4-one, 2,3-dihydro-3,5-dihydroxy-6-methyl; 4. 1-Hexene,3,3,5-trimethyl; 5. Thymol; 6. Phenol,4-methoxy-2,3,6-trimethyl; 7. 5-Methyl-1-nitro pyrazole; 8. 2,6,10-Dodecatrien-1-ol,3,7,11-trimethyl (E,E); 9. 2-Naphthalenemethanol, decahydro-alpha.alpha.,4a-trimethyl-8-methylene-,[2R-(2.alpha.,4a.alpha.,8a.beta.)] 10. Tetradecanoic acid; 11. 2-Pentadecanone,6,10,14-trimethyl; 12. Hexadecanoic acid, methyl ester; 13. n-Hexadecanoic acid; 14. 9,12-Octadecadienoic acid, methyl; 15. 8-Octadecenoic acid, methyl ester, (E); 16. 9-Octadecenoic acid, (E); 17. Bicyclohexyl-2,3′-dione; 18. 9,17-Octadecadienal, (Z); 19. Cholest-4-en-3-one.

The chromatogram of the standard mixture and chemical composition of BCho is presented in Figure 1. The peak with the number 4 is the solvent used in the standard mixture.

### 3.2. Percentages of 5-hydroxymethylation (5-hmC) in the Whole Blood from Ewes during Different Stages of Gestation and Percentages of 5-hydroxymethylation (5-hmC) of Lambs 35 Days after Birth

There was no difference among the treatments for the percentages of 5-hmC before conception. At 50 days after conception, T1 led to a greater percentage of 5-hmC (*p* < 0.05) (Table 2). T4 also led to a greater percentage of 5-hmC during the first third of pregnancy, and T2 and T3 maintained the highest percentages of 5-hmC (*p* < 0.05) (Table 2). The percentages of 5-hmC DNA were greater in offspring born to T2 (*p* < 0.05) compared with the other treatments (Table 2).

### 3.3. Nutritional Programming Analyses of Ewes and Offspring Supplemented with Herbal Choline during Different Stages of Gestation

There were no statistically significant differences for the birth weight, initial weight, final weight, and live weight changes in the ewes supplemented with BCho. Additionally, there were no statistically significant differences among the groups for birth lamb weight, weaning lamb weight, or daily weight gain (Table 3).

### 3.4. Milk Quality

The colostrum protein, fat, lactose, and solids-not-fat concentrations were greater in the ewes supplemented with BCho throughout gestation (*p* < 0.05). Similarly, during the first five weeks of lactation, supplementation with BCho throughout gestation resulted in greater contents of protein, fat, lactose, and solids-not-fat (*p* < 0.05) (Table 4).

## 4. Discussion

Nineteen organic compounds were detected in BCho, some of which contain methyl groups. Previous studies [27,28] provide solid evidence that a number of the compounds in BCho serve as methyl donors and modify the methylation status of DNA—e.g., hexadecenoic acid methyl ester (C17:0), octadecenoic acid methyl ester (C18:1 *cis-9*; C18:1 *cis-8*), and thymol.

Hexadecenoic acid methyl ester (C17:0) and the isomers of octadecenoic acid (C18:1 *cis-9*; C18:1 *cis-8*) have been reported as the most abundant monounsaturated fatty acid in nature. Iijima [29] reported that C18: 1 represents between 20 and 30 percent of lipids in many animal tissues. Beeharry et al. [30] described the role of C17:0 as a potent molecule that protects DNA from oxidative damage and apoptosis. Some reports have associated fatty acids methyl esters (FAME) with the DNA methyl groups added [31,32]. Jukic et al. [33] reported that thymol obtained from essential oils in an aromatic plant (*Thymus vulgaris* L.) inhibited acetylcholinesterase activity in vitro. Thus, some of the observed effects when BCho was fed could be due to thymol promoting acetylcholine recycling—i.e., providing methyl groups for acetylcholine formation and the subsequent methylation of DNA [32,33].

As described previously, feed intake is the main source of free methyl donors, and methyl donor supplementation modifies the methylation status of DNA by participating in a cellular process of the one-carbon metabolism [21,32]. Based on the BCho characterization, BCho could provide labile free methyl groups for DNA methylation. Henning et al. [34] described the effects in DNA methylation by green tea, concluding that herbal compounds have the ability of epigenetic alterations due to the donation of methyl groups. Additionally, in mammals Shahrajabian et al. [35] concluded that herbal compounds could affect the levels of genomic DNA methylation and demethylation during different stages of age. In other studies [34], the evidence is not solid, probably due to the frequency and form of herbal consumption, which likely introduced some measurement error.

The percentages of 5-hmC in the whole blood from ewes that were supplemented increased during the first 50 days of supplementation with BCho. Masala et al. [36] reported that 5-hmC, as a transient mechanism during demethylation in growing oocytes, together with stable percentages of methylation hinted at an additional biological role similar to active demethylation and hydroxymethylation of the genome. In humans, it has been demonstrated that supplements cataloged as methyl donors in the daily diet can increase or modify the percentages of 5-hmC during different stages of pregnancy. Pauwels et al. [21] reported increases in the percentages of 5-hmC, with a high intake of methionine pre-pregnancy. In contrast, during the first trimester the percentages of 5-hmC were lower. Choline, a betaine, intake in the first week was negatively associated with hydroxymethylation [23]. These nutrients (methionine, choline, and betaine) are considered the main players in the one-carbon metabolism pathway—i.e., the methionine cycle, folic acid cycle, and transsulfuration. A higher rate of transsulfuration has been observed in early gestation and a higher rate of transmethylation in the third trimester [23,37]. The increase in the percentage of 5-hmC during the first 45 days of supplementation and throughout gestation in the present study could be associated with the amount of methyl groups consumed through BCho.

The percentages of 5-hmC in offspring whole blood from ewes supplemented with BCho during the second third of gestation were the greatest. This response could have been associated with the transfer of methyl groups from the ewe to the fetus. In humans, it has been reported that the increase in dietary methyl donors consumed alters the trajectories of DNA methylation during pregnancy [38]. The increase in the percentages of 5-hmC in the lambs born to dams with supplementation during the second third of gestation could have been associated with the amounts of dietary methyl groups and could have affected organ development (brain and liver) and increased the hereditable capacity of 5-hmC in whole blood [39]. Wu and Zhang [40] reported that the methylation of DNA could occur throughout gestation and concluded that differences in methylation of whole blood from newborn is in response to dietary methyl donor intake of the mother. During the second and third trimester of pregnancy, the supplementation with methyl donors results in significant changes in DNA methylation in the cord and peripheral blood [41]. However, other studies suggested that biologically, methylation, demethylation, and probably hydroxymethylation are very similar between the second and third trimester in humans [42].

In recent years, researchers have studied the importance of the placenta in terms of changes in DNA methylation and pregnancy complications. In addition to altering DNA methylation patterns in the fetus, the dietary methyl donor intake during pregnancy may also influence methylation patterns in the placenta. The principal factor determining intrauterine growth rate is the supply of nutrients from the placenta to the fetus, which depends on placental morphology, size, and blood supply. Green et al. [43] suggested that the most important role of 5-hmC in placenta is the association between gene expression and genomic demethylation; nevertheless, Mitsuya et al. [44] considered that placenta could regulate the DNA methylome when hydroxymethylation was enriched for pregnancy. This regulation is linked to an altered DNA methylome that may affect placental gene expression in relation to offspring development.

For the analysis of nutritional programming, the data obtained for the percentages of 5-hmC in whole blood from ewes supplemented in the first 45 days and throughout gestation relative to ewes supplemented during the second third of gestation, that the development of the lambs would be different; however, the lamb birth weight, lamb weaning weight and daily weight gain were not statistically significant among the treatments. Thus, the data from the current experiment suggest that modifications in 5-hmC of whole blood may not be related with growth performance in the offspring. There are few articles published that correlated the consumption of methyl groups during pregnancy with offspring growth. O’Neill et al. [45] indicated that supplementation during pregnancy with methyl donors may not lead to similar epigenetic effects across target organs, tissues, whole blood, and or cell types in the offspring [46]; however, O’Neill et al. [45] and McGee et al. [37] suggested that although the donation of methyl groups to DNA may not impact offspring birth weight, it could alter gene regulation and decrease the risk of metabolic diseases during early life and adulthood.

Chandra et al. [47] reported that supplementation with herbal compounds with active substances affect mammary gland secretory cells, with a subsequent increase in milk production and changes in the content of solids-not-fat and protein. Supplementation during all gestation with BCho increased milk production. We hypothesized that the milk quality was going to be modified by the bioactive compounds in BCho, which would increase colostrum quality. Although there is evidence in sheep [12] that supplementation with 4 gd^−1^ of BCho 30 d after to 30 d before parturition increased the milk yield and lamb weight at birth, it did not affect the milk components (protein, lactose, fat, non-fat solids, and total milk solids). Holstein cows that received 15 gd^−1^ of BCho for 90 days after calving had an increased in milk production by 3% [16]. The same herbal product was evaluated in different doses (0, 10, and 20 gd^−1^/cow) in dairy cows for 90 days [48] and showed a linear response in milk production. As observed in our study, there is a positive association for colostrum and milk quality with the supplementation of BCho throughout gestation.

## 5. Conclusions

This experiment allowed us to detect the effects of BCho inclusion during the entire gestation on DNA hydroxymethylation in sheep whole blood cells. Significant changes in the percentages of 5-hmC in whole blood during the different stages of gestation could be attributable to the herbal formulas containing bio-active substances, with a possible role in adaptation to pregnancy and lactation. The supplementation with BCho had no impact on the weight at birth and the development growth in offspring, but it increased the milk yield and some quality parameters of colostrum and milk. Future research should focus on elucidating the relationship among varying intake patterns of herbal formulas during pregnancy, the epigenetic effects, and the subsequent health outcomes in the adult offspring.

## Figures and Tables

**Table 1:** Chemical composition (% dry matter (DM) basis) of the corn silage fed to the pregnant ewes during gestation.

Item	Percent (%)
Dry Matter	38.83
Crude Protein	12.90
Neutral detergent fiber	40.10
Acid detergent fiber	29.70
Calcium	0.68
Phosphate	0.34
Ash	9.28
Ether extract	3.86

**Table 2:** Percentages of 5-hydroxymethylation (5-hmC) in the whole blood from ewes supplemented with herbal choline at different stages of gestation.

Days of Pregnancy	Treatments					SEM	p-Values
	First Third	Second Third	Last Third	Throughout Gestation	Control		
Before conception—10 day	0.055 ^a^	0.060 ^a^	0.058 ^a^	0.061 ^a^	0.054 ^a^	0.0081	0.36
50 day	0.064 ^a^	0.055 ^b^	0.035 ^c^	0.079 ^a^	0.024 ^c^	0.0082	0.01
100 day	0.054 ^b^	0.053 ^b^	0.036 ^c^	0.060 ^a^	0.049 ^bc^	0.0081	<0.01
150 day	0.030 ^b^	0.037 ^b^	0.028 ^b^	0.047 ^a^	0.034 ^b^	0.0080	<0.01
Lamb at 35 day after birth	0.037 ^b^	0.075 ^a^	0.038 ^b^	0.036 ^b^	0.027 ^b^	0.005	<0.01

SEM, standard error of the mean; ^a–c^ Means within a row with different superscripts differ (*p* < 0.05).

**Table 3:** Nutritional programming analyses of lambs during the first 5 weeks of age.

Item	Treatments					SEM	*p*-Values
	First Third	Second Third	Last Third	Throughout Gestation	Control		
Lactating Ewe							
Ewe birth weight (kg)	61.66	60.33	60.66	61.33	64.00	4.63	0.96
Initial lactating weight (kg)	53.33	51.66	48.33	51.00	52.66	13.65	0.33
Difference (kg)	0.238	0.274	0.352	0.295	0.323	0.91	0.14
Final lactating weight (kg)	8.33	8.66	12.33	10.44	11.33	6.34	0.22
Live weight changes (kg)	8.33	8.66	12.33	10.44	11.33	6.34	0.22
Offspring							
Birth lamb weight (kg)	5.66	5.84	4.83	6.27	4.62	0.54	0.32
Weaning lamb weight (kg)	13.55	12.49	11.98	12.35	11.52	0.97	0.33
Daily weight gain (g)	225	190	204	173	196	0.20	0.21

SEM, standard error of the mean.

**Table 4:** Effect of the supplementation with BCho during the different stages of gestation on the quality of colostrum and milk.

Item	Treatments					SEM	*p*-Values
	First Third	Second Third	Last Third	Throughout Gestation	Control		
Milk production (mL)	758.67 ^c^	1016.67 ^ab^	713.33 ^c^	1142.67 ^a^	907.33 ^bc^	14.05	<0.01
Colostrum Quality, %							
Protein	6.57 ^b^	4.43 ^c^	6.86 ^b^	8.42 ^a^	4.27 ^c^	0.55	<0.01
Fat	4.23 ^c^	8.47 ^b^	10.53 ^b^	13.53 ^a^	8.45 ^b^	2.39	<0.01
Lactose	8.31 ^ab^	7.78 ^b^	10.42 ^ab^	11.66 ^a^	7.28 ^b^	5.06	<0.01
Solids-not-fat	12.83 ^b^	14.16 ^ab^	18.76 ^ab^	20.59 ^a^	13.26 ^b^	7.45	<0.01
Milk Quality, %							
Protein	3.22 ^b^	3.26 ^ab^	3.05 ^c^	3.34 ^a^	3.33 ^a^	0.01	0.01
Fat	6.32 ^ab^	6.02 ^ab^	5.69 ^b^	6.61 ^a^	5.59 ^b^	0.18	0.01
Lactose	4.95 ^a^	4.86 ^a^	4.61 ^b^	4.97 ^a^	4.96 ^a^	0.05	0.02
Solids-not-fat	8.87 ^a^	9.05 ^a^	8.25 ^b^	9.04 ^a^	8.92 ^a^	0.11	0.01

SEM, standard error of the mean; ^a–c^ Means within a row with different superscripts differ (*p* < 0.05).
